# Detection of Intraepithelial and Stromal Langerin and CCR5 Positive Cells in the Human Endometrium: Potential Targets for HIV Infection

**DOI:** 10.1371/journal.pone.0021344

**Published:** 2011-06-29

**Authors:** Tove Kaldensjö, Pernilla Petersson, Anna Tolf, Gareth Morgan, Kristina Broliden, Taha Hirbod

**Affiliations:** 1 Department of Medicine, Division of Infectious Diseases, Center for Molecular Medicine, Karolinska Institutet, Karolinska University Hospital, Stockholm, Sweden; 2 Department of Laboratory Medicine, Division of Pathology, Karolinska Institutet, Karolinska University Hospital, Stockholm, Sweden; 3 Department of Clinical Pathology and Cytology, Uppsala University Hospital, Uppsala, Sweden; Institut Pasteur, France

## Abstract

Both the upper (endocervix and uterus) and lower (ectocervix and vagina) female genital tract mucosa are considered to be target sites for sexual transmission of HIV. There are a few reports on the T cell and antigen-presenting cell distribution in human endometrial tissue however, there is little known about the expression of the HIV co-receptor CCR5 and HIV-binding C-type lectin receptors on endometrial cell subsets. We therefore assessed endometrial tissue sections from HIV seronegative women undergoing hysterectomy of a benign and non-inflammatory cause for phenotypic characterization of potential HIV target cells and receptors by immunohistochemistry. Langerin was expressed on intraepithelial CD1a+CD4+ and CD11c+CD4+ Langerhans cells. Furthermore, CCR5+CD4+CD3+ T cells, DC-SIGN+MR+CD11c+ myeloid dendritic cells and MR+CD68+ macrophages were found within or adjacent to the epithelium of the uterine lumen. In addition, occasional CD123+ BDCA-2+ plasmacytoid dendritic cells were detected deep in the endometrial stroma. Both T cells and several antigen-presenting cells were detected in lymphoid aggregate formations in close proximity to the epithelial lining. The finding of intraepithelial and stromal Langerin+ cells as well as CCR5+ CD4+ T cells is novel for human endometrium.

## Introduction

The female genital tract is responsible for two complex tasks; creating an environment for fertilization and protection against potential pathogens. As all other mucosal sites the female genital tract is exposed to a wide range of pathogens, not the least sexually transmitted infections such as the human immunodeficiency virus type 1 (HIV). The uterus and the endocervix are protected by the cervical mucus, which contains numerous antiviral innate immune molecules as well as constitutes a physical barrier. Despite this, sperm and radioactive microspheres enter the uterus within minutes after placement in the vagina [1], [2]. Semen from an HIV positive individual contains free virions and infected leucocytes. In addition, spermatozoa can capture HIV and transmit the virus to dendritic cells (DCs) [3]. Thus, the DC population may together with other target cells contribute to initial HIV transmission events in the endometrium and therefore needs further characterization.

Even when intact, the single layer columnar epithelium lining the endocervix and uterus is a less robust barrier to pathogens as compared to the multilayered squamous epithelium lining the ectocervix and vagina. Thus, invading pathogens may more easily access potential mucosal HIV target cells such as CD4+ T cells, DCs and macrophages. In addition to CD4+ T cells, which have previously been identified in human endometrial tissue [4], [5], [6], Langerhans cells (LCs), interstitial DCs and macrophages are all target cells for HIV. These cells can bind the virus through conventional binding to CD4 and the main co-receptor CCR5 as well as other receptor pathways including C-type lectin receptors (CLRs) [7]. Little is known about the presence of CLRs such as the mannose receptor (MR) [8], [9], the DC-specific intercellular adhesion molecule-grabbing intergrin (DC-SIGN) [10] and Langerin [11] in the human endometrium. The relative importance of primary HIV entry and infection through the different pathways is affected by the location, timing and concentration of the inoculum and host factors. For example, under non-inflammatory conditions immature LCs may form a barrier against HIV infection by efficiently capturing and degrading the virus through Langerin, rather than binding HIV and subsequently transferring the infectious virus particles to other target cells [12].

In this report we characterize potential HIV target cells and cellular receptors in the human endometrium, which constitutes an accessible site to incoming pathogens upon sexual intercourse [13].

## Material and Methods

### Study population and sample collection

Endometrial tissue sections from the uterine corpus were obtained from eight women undergoing hysterectomy for non-malignant and non-inflammatory indications (leiomyomata and adenomyosis); mean age was 48 years (range 39–52 years). The inclusion criteria were: HIV IgG seronegative, no clinical symptoms of sexually transmitted infections during the prior three months and no use of hormonal therapy. The hysterectomy samples were immediately transported on ice to the pathology department where a pathologist specializing in gynecological specimens collected endometrial tissue sections. A blinded pathologist determined all samples for menstrual cycle stage by endometrial dating. Two study subjects were in the proliferative phase, three were in the secretory phase and three had an inactive endometrium. Written informed consent was obtained from all study subjects and ethical approval was obtained from the Regional Ethical Review Board in Stockholm.

### In situ detection of cellular markers by immunostaining and confocal microscopy

The endometrial tissue sections were processed and snap frozen in liquid nitrogen within 30 minutes of surgical removal. Following previous protocols [14], [15] the cryopreserved tissue samples were sectioned to 8 µm and fixed in 2% formaldehyde. Selected anti-human monoclonal and polyclonal antibodies were used to detect the immune markers of interest: Langerin (clone: AF2088), DC-SIGN (clone: 120507) DLEC (BDCA-2 clone: AF1376) (R&D systems, Minneapolis, MN) MR (clone: 19.2) CD11c (clone: B-ly6) CD123 (clone: 9F5), CD123 (clone: 7G3), HLA-DR (clone: L243), CD4 (clone: SK3), CD8 (clone: SK1), CD3 (clone: SK7) (Becton Dickinson, Franklin Lakes, NJ) CD1a (clone: NA/1/34-HLK) (Serotec, Düsseldorf, Germany), CD68 (clone: EBM11) (DAKO, Stockholm, Sweden) and CCR5 (clone: MC-5) (kindly provided by Professor M. Mack from the University Clinic of Regensburg, Germany). The staining reactions were developed brown by using diaminobenzidine tetrahydrochloride (DAB; Vector Laboratories, Burlingame, CA) and nuclear counterstaining was performed with hematoxylin. Digital images were transferred from a DMR-X microscope (Leica, Wetzlar, Germany) into a computerized image analysis system, Quantimet, Q 550 IW (Leica Imaging Systems, Cambridge, UK). For 1 and 2-color fluorescent staining, combinations of relevant antibodies were used followed by the appropriate Alexa Fluor–conjugated secondary antibody. Fluorescently labeled cells were evaluated using the Qwin 550 software and a filter-free spectral confocal microscope (Leica TCS SP2 AOBS). Negative control staining consisted of irrelevant mouse, rat or goat IgG (Dako).

### Quantitative analysis

**Figure 1:**
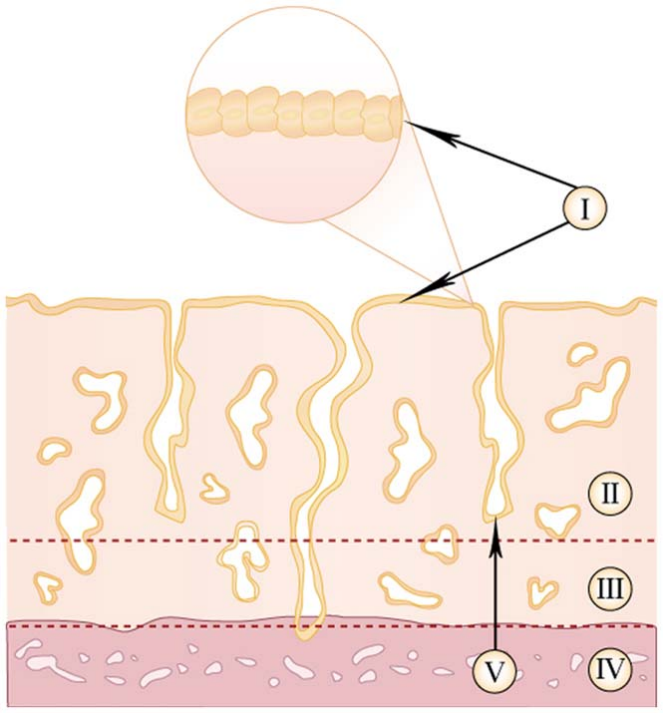
Schematic picture of the uterus. Schematic picture showing the myometrium and the different components of the human endometrium: I: luminal epithelium; II: lamina superficialis; III: lamina basalis; IV: myometrium and V: glandular epithelium. Quantitative assessment of endometrial tissue samples was performed in all sections but the myometrium.

Expression of the markers of interest was analyzed in a median of 1.9×10^7^ µm^2^ endometrial tissue section per individual (range 1.3–2.4×10^7^ µm^2^), including the: luminal epithelium; lamina superficialis; lamina basalis and the glandular epithelium (Figure 1). The myometrium was excluded from all analysis. All calculations were performed manually in Adobe Photoshop CS3 (Adobe Systems Incorporated, San Jose, CA) and all tissue sections were scanned and assessed in duplicates with result variations less than 10%.

## Results

**Figure 2:**
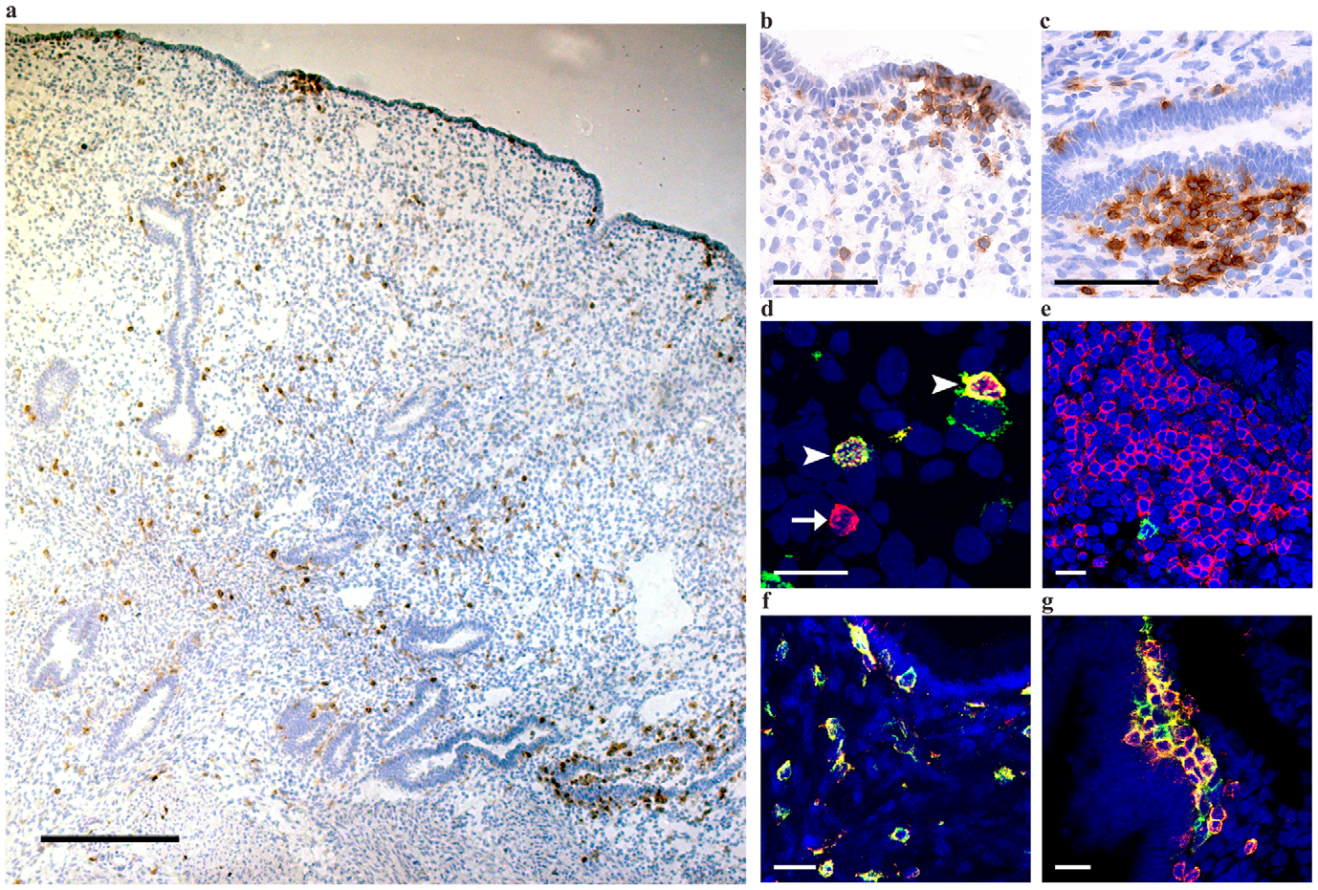
Endometrial CD4+cells express CCR5. (a) Scattered CD4+ cells were detected in the luminal and glandular epithelium as well as both stromal locations (lamina superficialis and lamina basalis). (b) Clusters of CD4+ cells were also detected just beneath the luminal epithelium and (c) adjacent to glandular epithelium. (d) A majority of the scattered CD4+ cells (red) co-expressed (yellow) CD3+ (green). (e) The lymphoid aggregate formations contained vast numbers of CD3+ cells (red) and occasional CD1a+ cells (green). (f) A positive co-localization (yellow) of CCR5+ cells (red) and CD4+ cells (green) was frequently observed both in the luminal epithelium and underlying stroma and (g) in the lymphoid aggregate formations. Arrowheads indicate example of double-positive staining; arrows indicate single-positive staining. Scale bars: a: 500 µm; b, c: 100 µm; d–f: 25 µm.

CD4 is considered to be the main receptor for HIV and is commonly expressed on T-cells (here defined as CD3+) but also on antigen-presenting cells. In this study, scattered CD4+ cells were detected throughout the endometrial epithelium and stroma of all study subjects (n = 8) (median: 0.005 positive cells/100 µm^2^ tissue [range: 0.003–0.009 positive cells/100 µm^2^ tissue]) and 3–22% of these cells were either detected within or adjacent (≤50 µm) to the luminal epithelium (Figure 2). Also, clusters of CD4+ cells were detected in lymphoid aggregate formations adjacent to the luminal and glandular epithelium of all study subjects (median size of aggregate: 1.7×10^4^ µm^2^ [range: 0.2–4.9×10^4^ µm^2^]). Approximately 30–50% of the scattered CD4+ cells were CD3+ T cells, whereas 100% of the CD4+ cells in lymphoid aggregate formations were CD3+ T cells (Figure 2). CCR5, the main co-receptor for HIV, was expressed on scattered CD4+ cells, CD4+ cells in lymphoid aggregates (Figure 2) and on CD3+ cells (data not shown).

**Figure 3:**
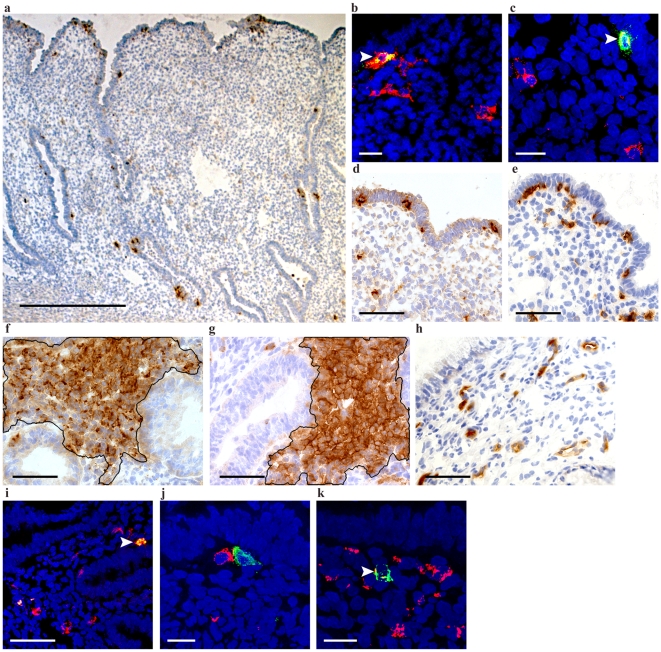
Distribution of antigen-presenting cell subsets in human endometrium. (a) CD1a+ cells were rare and mainly found within the luminal or glandular epithelium. (b) CD1a+ cells (green) co-expressed (yellow) CD11c+ (red), although not all CD11c+ cells expressed CD1a. (c) The CD1a+ cells (green) also expressed CD4 (red). (d) CD11c+ and (e) CD68+ cells were present in much higher frequencies in both the epithelium and stroma and clusters of both (f) CD11c+ and (g) CD68+ cells were present in the lamina basalis adjacent to glandular epithelium (marked area: 7.1×10^4^ µm^2^ and 8.1×10^4^ µm^2^, respectively). (h) CD123+ cells were solely present in the stroma as scattered cells and in streak formations. (i) Some but not all CD123+ (red) cells co-localized (yellow) with BDCA-2 (green). BDCA-2+ (green) neither co-localized with (j) CD11c+ (red) nor (k) CD68+ (red) cells, although they were occasionally detected in close proximity to each other. Arrowheads indicate example of positive staining. Scale bars: a: 500 µm; b, c, j, k: 25 µm; d–i: 100 µm.

Antigen-presenting cells can express HIV-binding CLRs that may facilitate viral transmission across mucosal surfaces. The presence of such cells was thus characterized in the endometrial tissue samples. LCs, macrophages, myeloid DCs (mDCs), and plasmacytoid DCs (pDCs) were here defined as CD1a+; CD68+; CD11c+ and CD123+BDCA-2+ cells, respectively. CD1a+ cells were rare (median: 0.0003 positive cells/100 µm^2^ tissue [range: 0.0001–0.001 positive cells/100 µm^2^ tissue]) and mainly detected within the luminal (40–60%) or glandular (45–60%) epithelium (Figure 3). The CD1a+ cells co-expressed CD11c and CD4 (Figure 3) and were occasionally detected within stromal CD4+CD3+ lymphoid aggregates (Figure 2). CD68+ and CD11c+ cells were more abundant (median: 0.009 and 0.008 positive cells/100 µm^2^ tissue, respectively [range: 0.006–0.01 and 0.007–0.01 positive cells/100 µm^2^ tissue, respectively]). Both CD68+ and CD11c+ cells were detected within the luminal epithelium (11–18% and 18–35%, respectively) and in stromal lymphoid aggregate formations (Figure 3). A subset of CD11c+ cells co-expressed CD4+ (data not shown). CD123+ cells were solely present in the stroma, both as individual scattered cells and organized in streak formations. CD123 is expressed by pDCs but may also be expressed by additional cell types, thus a supplementary pDC-specific marker, BDCA-2, was used. There was a vast difference in the number of CD123+ and BDCA-2+ cells (median: 0.006 and 0.0002 positive cells/100 µm^2^ tissue, respectively [range: 0.002–0.008 and 0.0001–0.0004 positive cells/100 µm^2^ tissue, respectively]), however, all BDCA-2+ cells were CD123+ (Figure 3). BDCA-2+ cells were distinctly diverse from all other investigated cell populations, although they were occasionally detected in close proximity to each other.

**Figure 4:**
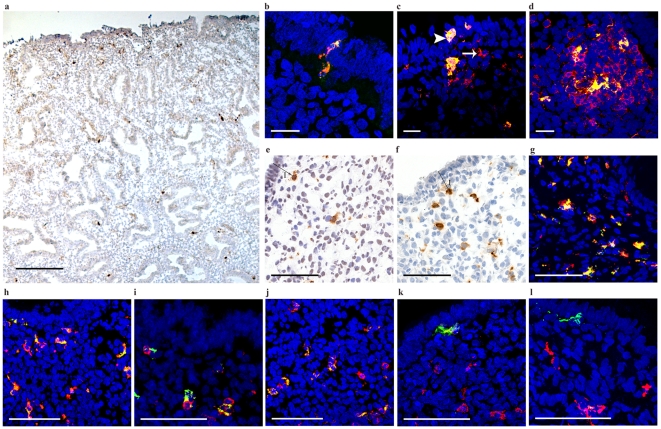
CLR expression on various DC subsets present in the endometrium. (a) Langerin positive cells were mainly detected within the luminal and glandular epithelium. Positive co-localization (yellow) was detected with Langerin+ cells (green) and (b) CD1a (red) and (c) CD11c (red). (d) Double-positive cells (yellow) expressing Langerin+ (green) and CD4+ (red) cells were also detected within CD4+ lymphoid aggregates. (e) DC-SIGN and (f) MR were detected in the lamina superficialis, just beneath the luminal epithelium (I: minimum distance of cell body to endometrial lumen: 49 µm and 40 µm, respectively). Both (g) DC-SIGN (green) and (h) MR(green) were co-expressed (yellow) on CD68+ cells (red) as well as (i and j, respectively) CD11c+ cells (red). Neither (k) DC-SIGN (red) nor (l) MR (red) were expressed on CD1a+ (green) cells. Scale bars: a: 500 µm; b–d: 25 µm; e–l: 100 µm.

Although HIV efficiently binds to CD4 and CCR5, the virus can also bind to CLRs including Langerin, DC-SIGN and MR. Langerin+ cells were distributed throughout the endometrial tissues of all study subjects (median: 0.003 positive cells/100 µm^2^ tissue [range: 0.002–0.009 positive cells/100 µm^2^ tissue]) and 11–39% of these cells were either detected within or adjacent (≤50 µm) to the luminal epithelium (Figure 4). The Langerin+ cells expressed CD1a, CD11c, and CD4, but not CD68 and were detected within stromal lymphoid aggregates (Figure 4). Neither DC-SIGN nor MR were detected within the luminal epithelium, although both CLRs were detected in close proximity (minimum distance to lumen: 49 µm and 40 µm, respectively). Both DC-SIGN and MR were expressed on CD68+ and CD11c+ cells but not on CD1a+ cells (Figure 4). The CD123+BDCA-2+ cells did not express any of the investigated CLRs (data not shown).

## Discussion

In this report we have demonstrated the presence of intraepithelial Langerin, CCR5 and CD4 expressing cells in human endometrium. These molecules are known HIV receptors and could thus serve as potential HIV-binding targets. Furthermore, the presence of stromal lymphoid aggregates, containing cells expressing Langerin, CCR5, CD4, CD11c and CD68 could hypothetically constitute a site for HIV replication as well as a functional site for local immune responses. Although such aggregates have previously been described in endometrial [4], [16], [17], [18], [19] and ectocervical tissue [20], but not in other parts of the human female genital tract under non-inflammatory conditions, the presence of potential HIV receptors such as Langerin have not. Previous findings of CCR5 expression in human endometrium [21] and uterine epithelial cells [22] have now been extended with phenotypic characterization in both the intraepithelial and stromal compartment.

The CD1a+CD4+CD11c+CD68- LC phenotype has been found at other mucosal sites [23] whereas single layer columnar epithelium has been proposed to lack Langerin+ LCs [24]. Yet, occasional CD1a+ cells have been detected in human first trimester deciduas [25] and non-pregnant endometrium [4], [26]. By staining for Langerin in freshly frozen tissues from non-pregnant women with non-inflammatory underlying conditions, we suggest that LCs can be constitutively expressed in the endometrial columnar epithelium, however their role in HIV transmission events at this site remain to be established. The conflicting results regarding the presence of rare CD1a+ LCs may be due to different staining techniques, amount of tissue investigated and type of study subjects. In addition to CCR5+CD4+CD3+ T-cells and Langerin+CD1a+CD11c+ LCs, additional HIV target cells including DC-SIGN+MR+CD11c+ mDCs, and MR+CD68+ macrophages were also widely distributed in the superficial layer of the endometrial stroma and may thus theoretically be accessible for HIV-infection following sexual intercourse.

Scattered and streak-forming CD123+ cells were further found in the stroma and a small subgroup of the scattered cells co-expressed BDCA-2, a phenotype specific for pDCs. These cells did not express any of the investigated CLRs. To our knowledge this is the first time CD123+BDCA-2+ pDCs have been shown in human non-inflamed endometrial tissue although the number of cells was very low. By using immunohistochemistry we could determine the exact localization of these double-stained cells, specifically in relation to the endometrial lumen and thereby extend previous studies that used flow cytometry on tissue extracts from human deciduas [27]. As blood-derived pDCs down-regulate their BDCA-2 expression upon in vitro maturation [28], the detected CD123+ BDCA-2- cells may constitute mature pDCs or basophils [29]. pDCs have been implicated as important mediators of immune activity in response to virus exposure in the endocervix [30], a mechanism that would be interesting to explore also in the uterus.

It must be remembered that the type and number of immune related cells changes throughout the menstrual cycle and with underlying medication, hormonal contraceptive use, disease or pregnancy [5], [6], [26], [31], [32]. Furthermore, the regional variations in histology and the size of the uterus complicate adequate assessment of cell populations. The study individuals in this report represented three different stages of the menstrual cycle. A similar distribution of all investigated cell populations and receptors were observed within their respective endometrium, however due to the small size of the study groups no inter or intra-group statistical analysis was performed. Future studies need to expand each study group to statistically verify variations in immune cell populations. Nevertheless, the present demonstrations of Langerin+ LCs, CCR5+ T cells and CD123+BDCA-2+ pDCs were novel observations at this anatomical site. The quantitative assessment showed a generally low number of the investigated cellular markers in the endometrial stroma. This was especially true for the BDCA2+CD123+ cells and the LC associated marker CD1a. In comparison with the immune cell numbers in the squamous cervicovaginal epithelium [31] the endometrial stroma exhibits a relative paucity of immune cells. However, this discrepancy must be considered with the regional differences in histology and function in mind.

The major site of HIV penetration into the female genital tract mucosa following vaginal intercourse is not clear. As single layer epithelium may be more easily traversed by HIV compared to multilayer epithelium [33], and since semen can reach the uterus following sexual intercourse, the endometrium might serve as target for HIV. Indeed, HIV has been shown to infect cells and tissues from the uterus representing all stages of the menstrual cycle as well as postmenopausal women in ex vivo models [34]. Our findings of endometrial intraepithelial and stromal cells expressing HIV-binding receptors adds to earlier reports on the distribution of HIV target cells in human endometrium [22], ectocervical tissue [14], [32], [35] and vagina [31], [36], [37].
